# Evolutionary dynamics of copy number variation in pig genomes in the context of adaptation and domestication

**DOI:** 10.1186/1471-2164-14-449

**Published:** 2013-07-05

**Authors:** Yogesh Paudel, Ole Madsen, Hendrik-Jan Megens, Laurent AF Frantz, Mirte Bosse, John WM Bastiaansen, Richard PMA Crooijmans, Martien AM Groenen

**Affiliations:** 1 Animal Breeding and Genomics Centre, Wageningen University, De Elst 1, Wageningen, WD, 6708, The Netherlands

**Keywords:** Structural variation, Copy number variation, Next generation sequencing data, Read depth method

## Abstract

**Background:**

Copy number variable regions (CNVRs) can result in drastic phenotypic differences and may therefore be subject to selection during domestication. Studying copy number variation in relation to domestication is highly relevant in pigs because of their very rich natural and domestication history that resulted in many different phenotypes. To investigate the evolutionary dynamic of CNVRs, we applied read depth method on next generation sequence data from 16 individuals, comprising wild boars and domestic pigs from Europe and Asia.

**Results:**

We identified 3,118 CNVRs with an average size of 13 kilobases comprising a total of 39.2 megabases of the pig genome and 545 overlapping genes. Functional analyses revealed that CNVRs are enriched with genes related to sensory perception, neurological process and response to stimulus, suggesting their contribution to adaptation in the wild and behavioral changes during domestication. Variations of copy number (CN) of antimicrobial related genes suggest an ongoing process of evolution of these genes to combat food-borne pathogens. Likewise, some genes related to the omnivorous lifestyle of pigs, like genes involved in detoxification, were observed to be CN variable. A small portion of CNVRs was unique to domestic pigs and may have been selected during domestication. The majority of CNVRs, however, is shared between wild and domesticated individuals, indicating that domestication had minor effect on the overall diversity of CNVRs. Also, the excess of CNVRs in non-genic regions implies that a major part of these variations is likely to be (nearly) neutral. Comparison between different populations showed that larger populations have more CNVRs, highlighting that CNVRs are, like other genetic variation such as SNPs and microsatellites, reflecting demographic history rather than phenotypic diversity.

**Conclusion:**

CNVRs in pigs are enriched for genes related to sensory perception, neurological process, and response to stimulus. The majority of CNVRs ascertained in domestic pigs are also variable in wild boars, suggesting that the domestication of the pig did not result in a change in CNVRs in domesticated pigs. The majority of variable regions were found to reflect demographic patterns rather than phenotypic.

## Background

Linking genotypic variation to phenotypic variation is one of the most challenging aspects of contemporary genome research. While several studies have found that single nucleotide polymorphisms (SNPs) can have drastic effects on phenotype [1,2], these types of variation are unlikely to solely explain the large phenotypic diversity found at the inter and intra specific level. Recent genomic studies have shown that variations, other than SNPs, such as structural variations (SVs) also play a prominent role in phenotypic evolution [3].

Polymorphic SVs may lead to different copy number of specific genomic regions within a population. These regions are often called copy number variable regions (CNVRs) and can range from 50 bases up to several megabases (Mb) [4]. CNVRs constitute roughly 5-12% of the human genome [5,6] and have been recognized as a source of phenotypic variation including susceptibility to specific diseases [5-8]. Duplication of genic regions can also result in evolution of new genes and gene functions that can have a significant impact on phenotypes [9-13]. For example, duplication of the *CCL3L1* gene can protect an individual against contracting HIV and developing AIDS [14] and a partial duplication of the Slit-Robo Rho GTPhase-activating protein 2 gene (*SRGAP2*), some around 3 million years ago (mya), created a novel gene function associated with cognitive abilities in humans [3,15,16].

In domestic animals the best-known examples of traits that are affected by CNVRs pertain the animal exterior. For instance, a duplication of the agouti signaling protein gene (*ASIP*) in sheep results in a different pigmentation [17]. The duplication of a set of fibroblast growth factor (*FGF*) genes in dogs leads to a characteristic dorsal hair ridge [18]. A copy number gain of the region containing the *KIT* gene causes the dominant white/patch coat phenotype observed in different European pig breeds [19,20]. Thus, the association of CNVRs with distinct large effects in species that very recently have undergone strong phenotypic alteration, most notably domesticated animals in the past 10 thousand years, raises the question of how rapid phenotypic alteration may be related to (large) structural variation in genomes.

*Sus scrofa* (domesticated pigs and wild boars; family: Sudiae) diverged from other *Sus* species some 4 mya and started to spread, from Southeast Asia, into the rest of its currently natural occurrence across most of the Eurasia about 1.2 - 0.6 mya (Frantz LAF, unpublished observations). Such a large bio-geographic range will result in a wide range of local adaptation that, in part, may be related to CNVRs. Domestication can be seen as a long lasting genetic experiment [21], and in the case of pigs has been carried out on the same wild ancestral species independently at least once in Europe and once in Asia [22,23]. Independent domestication implies independent breeding practices in Europe and Asia for several thousand years. Historical records revealed that breeding was more intensive in Asia than in Europe for centuries [24]. Different breeding regime led to intensive trading of breeds between Europe and Asia, especially at the onset of the industrial revolution when Europeans massively imported Asian breeds [24,25]. Since the wild ancestor is still present throughout the entire natural range, among domesticated species, *Sus scrofa* provides a well suitable framework for studying effects of both adaptation and domestication on mammalian genome structure, such as CNVRs.

The recent completion of the porcine genome [25] and the advent of high-throughput sequencing methods, now allow for a comprehensive screen of variation, including structural variation in the pig. Although several different methods e.g. SNP arrays and array CGH have been applied to screen for SVs, methods based on next generation sequencing (NGS) technology in general, and read depth (RD) based methods [26] in particular, revealed better performance in detecting CNVRs. The advantage of this approach is seen especially in and near highly duplicated genomic regions, such as segmental duplications (SDs) where most of the array based methods fail [27,28].

In this study the RD method was applied on NGS data of 16 *Sus scrofa* individuals, representing the diversity of both wild and domesticated pigs, firstly to detect SVs/CNVs in the pig genome and secondly to relate the evolution of SVs/CNVs to pig genomics features and to population/domestication histories.

## Results

### Data selection, copy number detection and definition of multi copy regions

In this study, 16 pigs were selected to cover a broad representation of pig diversity of both wild and domestic pigs. The selection of samples included three wild boars from Asia and three from Europe and five domesticated individuals from Asia and five from Europe (Table 1; Additional file 1: Table S1A). Whole genome re-sequenced data were obtained for the 16 samples with the average coverage per sample varying between 7x and 11x. Reads were aligned against the porcine reference genome (*Sus scrofa* build 10.2 [25]) using mrsFAST [29]. The RD method [26] was used to detect copy numbers (CNs) in the 16 pig individuals (see materials and methods for details). From the estimated CN we defined regions of CN gains (termed multi copy regions (MCRs)) as regions ≥ 6 kilobases (Kb) and CN > 3. We detected 61,761 MCRs in the 16 individuals with individual numbers of MCRs ranging from 3,750 in an Asian domestic (AsD05) to 3,984 in a European wild boar (EuWB03). The average number of MCRs per individual was 3,860 covering 49.93 Mb (Table 1; Additional file 1: Table S1A). The size of the MCRs identified varied from the predefined minimum of 6 Kb to 122 Kb with an average size of 13 Kb. The majority of MCRs was found to be common in all 16 individuals. The number of MCRs that were found specific to single individual ranged from 0–12. Regions of CN loss were also identified, but we observed a positive correlation between sequence depth and regions of CN loss. With the used sequence coverage, this resulted in a considerable numbers of false positive CN losses (data not shown) and it was therefore decided to exclude CN losses from further analyses.

**Table 1:** **Number and total size of multi copy regions in the 16 individuals**^**1**^

**Region**	**Populations**	**Individual**^**1**^	**Sample**	**Read**-**depth**^**2**^	**Total MCR**	**Size (Mb)**
		AsWB01	Japanese WB	11	3764	48.9
	**Wild**	AsWB02	N. Chinese WB	10	3832	49.75
		AsWB03	S. Chinese WB	10.1	3953	51.23
**Asia**		AsD01	Meishan	9	3926	50.89
		AsD02	Meishan	9.1	3854	49.89
	**Domestic**	AsD03	Xiang	8.1	3858	49.74
		AsD04	Xiang	8	3861	50.19
		AsD05	Jianquhai	10.5	3750	47.99
		EuWB01	Dutch WB	9	3768	48.79
	**Wild**	EuWB02	Dutch WB	8	3816	49.2
		EuWB03	Italian WB	10	3984	51.47
**Europe**		EuD01	Large white	8	3909	50.59
		EuD02	Large white	8	3929	50.9
	**Domestic**	EuD03	Landrace	8	3800	48.85
		EuD04	Duroc	7.1	3814	49.54
		EuD05	Pietrain	11	3943	51.14

^**1**^More details on individual information (Additional file 1: Table S1A).

^**2**^Average read-depth of the diploid region.

### Copy number variable regions among pigs

**Figure 1:**
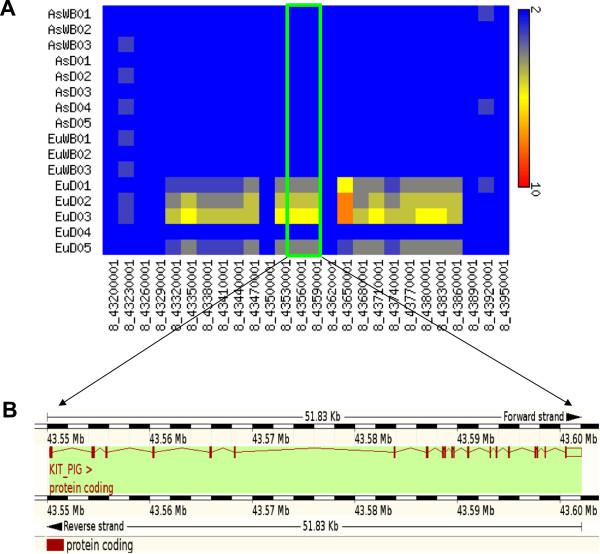
**Region in chromosome 8 with the *KIT *gene.** The region in chromosome 8 with *KIT* gene (SSC8: 43,550,236-43,602,062), which is responsible for dominant white color in pigs shows an increase in the number of copies in the European domestic individuals. **A)** Heatmap of the region containing the *KIT* gene. Blue color represents the diploid region where red color represents the region with copy number higher than 9. **B)** Location of the *KIT* gene in the porcine genome (extracted from Ensembl browser).

**Figure 2:**
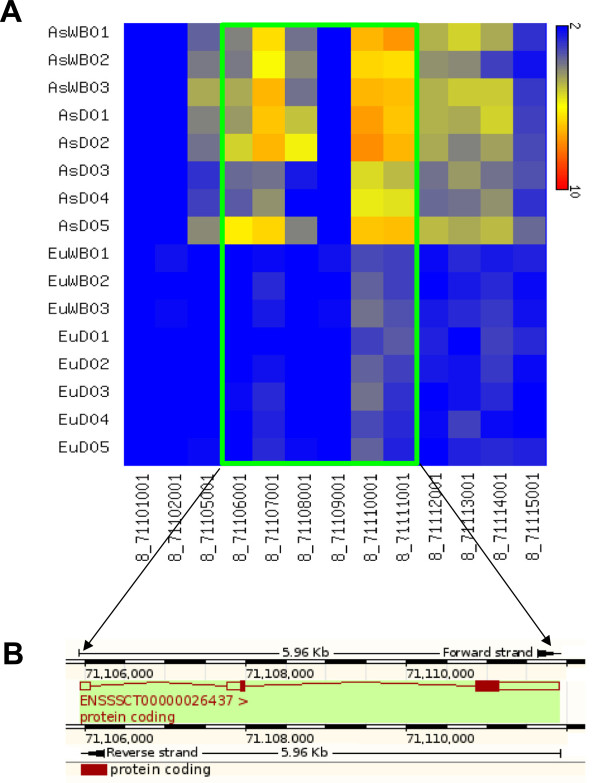
**The *UGT2B10 *gene in the porcine genome.** The *UGT2B10* gene, which is involved in detoxification, shows increased copy number in the Asian individuals. **A)** Heatmap showing higher copies of *UGT2B10* (ENSSSCG00000026944; SSC8: 71,105,942-71,111,905 ) in Asian individuals (CN 5 to 9). **B)** Location of the *UGT2B10* in the porcine genome (extracted from Ensembl browser).

CNVRs can be identified by comparing CN of the overlapping MCRs in different individuals. We identified 5,097 MCRs with their corresponding CN in the 16 individuals. The standard deviation (s.d.) of CN of each MCR was calculated and MCRs with a s.d. ≥0.7 among the 16 individuals were regarded as CNVRs. In total, 3,118 putative CNVRs were obtained with an average size of 13 Kb, comprising 39.72 Mb of the porcine genome (Additional file 2: Table S2A; See Figures 1, 2 and Additional file 3: Figures S2 and S3 for examples of CNVRs). The CNVR density per chromosome varies from 0.85% on chromosome 18 to 2.29% on chromosome 2 (Additional file 2: Table S2B).

### Experimental validation

We evaluated the accuracy of CNVRs prediction by quantitative real time-polymerase chain reaction (qPCR). Ten genic CNVRs, ten non-genic CNVRs and four diploid regions were randomly selected and tested using two distinct primer sets per locus. 23 of the 24 assays were successful and for those we found 100% agreement with our CNVRs predictions indicating a low false discovery call of CNVRs by the methodology and thresholds used in our analysis. Details of the qPCR primers can be found in Additional file 4: Table S4C. We also compared the predicted CNVRs with known CNVRs. The region in chromosome 8 containing the *KIT* gene in the pig genome, which is known to be copy number variable between different European breeds confirms our results [19,20] (Figure 1).

### Association of CNVRs with genomic features

**Figure 3:**
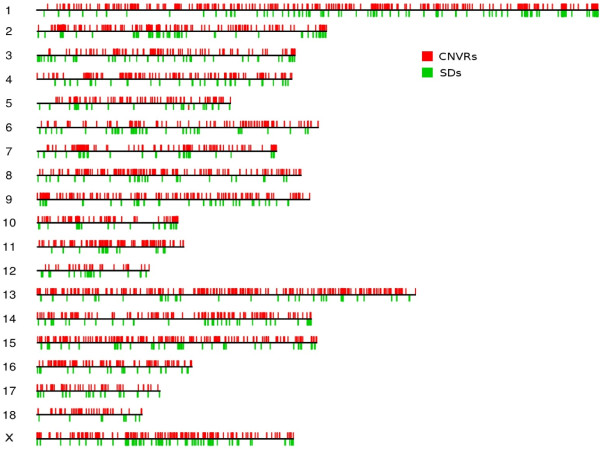
**Distribution of CNVRs and SDs across the porcine genome**. Black lines represent all 18 autosomes and the sex chromosome X. Red lines on the upper part of chromosomes indicate the 3,118 CNVRs and green lines on the lower part of chromosomes indicate 1,934 SDs.

Segmental duplications (SDs) (duplicated sequences larger than 1 Kb with more than 90% sequence similarity) act as promoter of CNVRs by facilitating non-allelic homologous recombination [30,31]. We compared the overlap between CNVRs with a list of 1,934 SDs previously identified in the pig genome [25]. We found that approximately 27.5% of SDs (533 out of 1934) were overlapping within the 10 Kb flanking region of CNVRs. Both the CNVRs and SDs appear to be non-randomly distributed across the genome (Figure 3). Highly repetitive sequences such as retrotransposons were also investigated for their correlation with CNVRs. The frequencies of major retrotransposon families were calculated by counting the number of bases of these elements in the 10 Kb flanking regions of CNVRs and SD separately (Table 2). We observed significant enrichments of LINE-L1 (P <0.001, Fisher test), LTR-ERV1 (P <0.001, Fisher test) and satellite elements (P <0.001, Fisher test) near CNVRs and SDs (Table 2).

**Table 2:** Densities of repetitive element families in pig CNVRs and SDs

**Repeats**	**PigCNVRs**^**1**^	**PigSDs**^**2**^	**Other intervals**^**3**^
Number of 10 Kb intervals	5304	2467	259660
LINE-L1	2872.95*	2852.95*	1368.88
LINE-L2	259.06	241.895	263.975
SINE-tRNA-Glu	1132.72	1133.05	1049.36
LTR-ERV1	248.19*	438.18*	148.055
LTR-ERVL-MaLR	170.467	183.131	159.755
SINE-MIR	193.498	209.735	233.435
DNA-hAT-Charlie	106.889	136.9616	111.46
Satellite	638.778*	576.016*	273.754

^1^Flanking 10 Kb regions of both end of CNVRs, all overlapping regions are merged.

^2^Flanking 10 Kb regions of SDs, all overlapping regions are merged.

^3^Whole genome is divided into 10 Kb regions.

*p-value (<0.001).

The guanine/cytosine (G/C) content of CNVRs and 10 Kb flanking region of CNVRs were assessed. Interestingly, it was observed that the G/C contents of CNVRs and 10 Kb flanking region of CNVRs are on average 1.5% and 1% lower than in the rest of the genome, respectively (Additional file 2: Table S2C).

### Functional analysis of copy number polymorphic genes

Genes overlapping with CNVRs were extracted and potential functional roles associated with CNVRs were identified by analyzing them. Although partial duplication of a gene can lead to a functional new gene, the likelihood that a gene is functional intuitively decreases with the fraction of a gene that is duplicated. To limit the false discovery rate caused by the inclusion of a large fraction of non-functional gene duplicates, we only considered genes which are at least 70% overlapping with a CNVR. Out of 21,627 genes annotated in the current genome build (*Sus scrofa* build10.2, Ensembl release 67 [25,32]), 575 protein-coding genes were found to overlap with the 454 CNVRs (14.56% of total CNVRs) (Additional file 5: Table S3A). A potential source of false positive calls are local high copy segments residing outside the gene exons resulting in CNVR calls without corresponding gene copy number variation. To avoid this type of false positives, the average depth of exon regions of the 575 genes, overlapping with a CNVR, were calculated (Additional file 5: Table S3A). Only genes with CN >2 in at least one individual and s.d. of ≥0.5 between 16 individuals were considered for further analysis. Of the 575 genes, 545 genes fulfilled this threshold (Additional file 5: Table S3B). Of the 11,629 one to one orthologous genes between human, cow and pig, only 25 were observed as multi copy genes including 10 olfactory receptor genes and genes like *KIT*, *BFAR*, *AHNAK* and *FLG2* (Additional file 5: Table S3C). Some of these genes only showed multiple copies in some of the individuals for example, *KIT* (Figure 1), whereas others showed high CN in all individuals like *FLG2* with CN ranging between 10–32.

The olfactory receptor gene family, one of the largest gene families in the porcine genome [25,33], is over-represented with 353 out of 545 genes overlapping with CNVRs (Additional file 5: Table S3D). Genes involved in immune response, for instance *IFN* (Alpha-8, 11, 14; Delta-2), *IFNW1*, *IGK* (*V1D*-*43*, *V2*-*28*, *V8*-*61*), *IL1B* and *PG3I*, were often observed as variable in CN between individuals. Defense related genes *NPG3* and *PMAP23*, which are specific to porcine genome, were found to be variable in CN. In addition, genes involved in metabolism, *AMY1A*, *AMY2*, *AMY2A*, *AMY2B* and *BAAT*, and detoxification, *ABCG2*, *UGT2B10*, *UGT1A3*, *CYPA11*, *CYPA22*, *CYP4F3* and *CYP4X1*, are also present in the list of copy number variable genes.

Few CN variable genes were observed to be unique to a specific group of pigs; Asian domestics, Asian wild boars, European wild boars or European domestic. One example is the genomic region at chromosome 8, which contains the *UGT2B10* gene (SSC8: 71105001–71116000; Additional file 5: Table S3A) and was found to have a high CN specifically in Asian domestics and Asian wild boars (Figure 2). Similarly, *BTN1A1*, *CDK17*, *CDK20*, *F5*, *FLG2*, *MGAT4C*, *RALGDS* and *SUSD4* show variation in CN in all individuals but have comparatively high CN in the Asian domestic individuals.

Human orthologs of the porcine genes were used to analyze the functional enrichment of genes affected by CNVRs. Gene ontology (GO) enrichment analysis revealed that most of these genes were involved in biological processes regulating sensory perception of smell (p < 0.001), signal transduction (p < 0.001), neurological process (p < 0.001) and metabolic process (p < 0.001) (Additional file 4: Table S4A).

### CNVRs between groups

**Figure 4:**
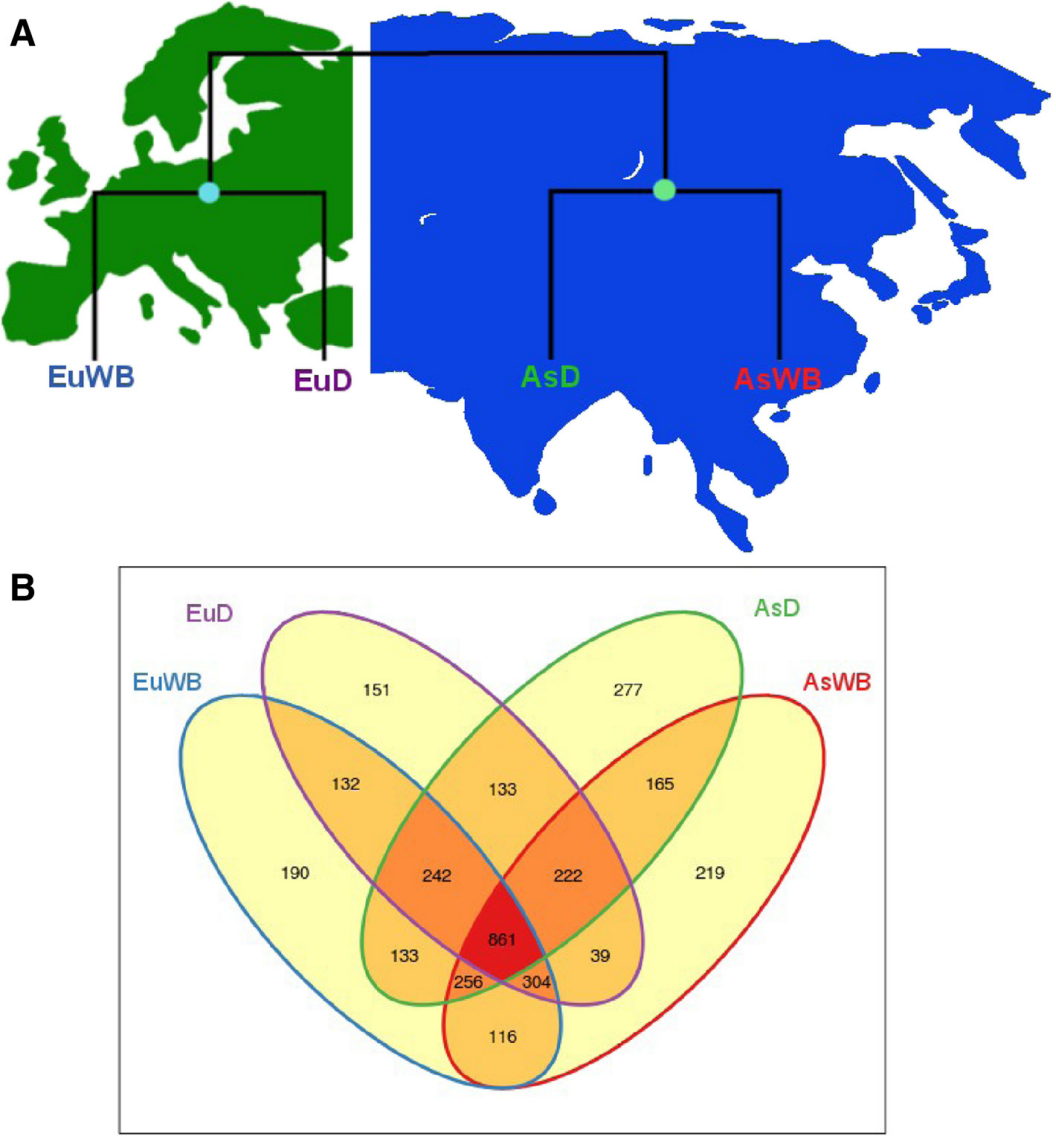
**Pairwise comparison between different groups. A)** Schematic representation of pigs across Eurasia. Two nodes shows two independent domestication events. **B)** Shared CNVRs between different populations.

The inclusion of pigs from the two independent domestications together with animals representing their wild ancestors enables preliminary investigation into whether the pattern of CNVRs was influenced by the process of domestication and/or the demographic history of pigs. For this particular comparison, to avoid any bias caused by sampling size, we included only 12 individuals, 3 from each of the 4 different groups based on their geographical origin/population (Asian wild, Asian domestic, European wild and European domestic) (Additional file 1: Table S1B). We compared the extent of overlap between the different groups and combination of the four groups and for each comparison, CNVRs were calculated separately (applying a threshold of ≥0.7 s.d. to call CNVRs) (Figure 4).

In all comparisons it is evident that the large majority of CNVRs are shared among the different groups (Figure 4B). The Asian group (including both Asian wild and Asian domestics) was found to have a higher CNVRs count (2,917) than the European group (2,779). Among the four groups, the Asian domestic group was found to have the largest number of CNVRs (2,289; of which 277 were group specific) with a ratio of 0.12 between shared and Asian domestic group specific CNVRs. The European domestic group was found to have the lowest number of CNVRs (2,084, 151 group specific) with a ratio of 0.07 between the shared and European domestic group specific CNVRs (Figure 4). Applying the same criterion as described above in the functional analyses, we extracted the genes overlapping with the CNVRs found in the comparative analyses. For each of the four groups we calculated the average cumulative count of genes and the s.d. of these overlapping genes (Additional file 4: Table S4B). It is notable that the number of genes situated in CNVRs seems to be higher in domesticated animals, both European and Asian, as compared to wild animals, but that the variation is lower in domesticated pigs compared to wild boars.

## Discussion

Pigs have been important in agriculture and welfare for thousands of years. The recent completion of a high-quality draft genome of *Sus scrofa*[25] enables the detailed investigation of a variety of genomics features. In this study, we used next generation sequence of 16 different wild as well as domestic pigs from Eurasia to generate a detailed map of CNVRs in the porcine genome.

### CNVRs in pig genomes (compared to other mammalian genomes)

We applied the read depth methodology [26,34,35] to estimate CNVRs. In total 3,118 CNVRs with an average size of 13 Kb were identified. Our result suggests that at least 1.5% (39.74 Mb) of the porcine genome can vary in CN of a size larger than 6 Kb, which is the minimum size we considered in this study. This figure is consistent with a recent study in cattle [35]. It is likely that the actual count and size of variable regions in the porcine genome is higher than our estimate. The stringent filtering criteria applied in our study, including a relatively high threshold of standard deviation to call a CNVR and exclusion of CN losses which were difficult to score with the sequence coverage currently available for the sampled individuals, likely inflated our false negetive discovery rate. In addition, 100% validiation of CNVRs tested by qPCR strengthens our confidence that our set of CNVRs is an underestimation rather than an overestimation.

Nevertheless, we estimated significantly more CNVRs than previously reported in pigs. Recently, two studies using array CGH inferred 259 CNVRs using 12 animals [36] and 37 CNVRs on chromosomes 4, 7, 14 and 17 in a set of 12 samples. In addition, three other studies using the Porcine SNP60 genotypes inferred 49 CNVRs using 55 animals [37], 382 CNVRs using 474 animals [38] and 565 CNVRs using 1693 pigs [39]. The limitations faced by these studies, may be related to different factors such as, homogeneous sampling (only domestic pigs), low marker density, non-uniform distribution of SNPs along pig chromosomes and/or a lack of specially designed non-polymorphic probes which is necessary to identify CNVR with higher resolution [40]. Here, the RD method based on next-generation sequencing, using 16 different wild as well as domestic pigs from Eurasia, resulted in a better resolution and higher confidence to call CNVRs. Thus, most of the CNVRs discovered in this study are novel relative to the previous studies and represents the largest catalog of porcine specific CNVRs to date.

### Association of CNVRs with genomic features

Previous studies suggested that repetitive elements play an important role in the formation of CNVRs and SDs [41]. Frequent breakage of DNA in and around the repeat regions could initiate non-allelic homologous recombination (NAHR) and result in CNVRs [42]. The enrichment of the repetitive elements LINE-L1, LTR-ERV1 and satellite elements at the boundaries of CNVRs and SDs in the porcine genome (Table 2), suggests that these families of repeat elements indeed facilitate the formation of CNVRs and SDs in the porcine genome. This is in accordance with the observation made by Giuffra *et al*. (2002), who has reported an association of LINE-L1 and the duplication of the region containing the *KIT* gene in the porcine genome [43]. Similarly, the slightly lower G/C content (1.5%) of CNVRs in the porcine genome suggests that the porcine CNVRs are likely to coincide with the gene-poor regions, which is consistent to the observation made in the human genome [44].

### Copy number polymorphic genes

In total, we found 545 genes overlapping with CNVRs representing a valuable resource for future studies on the relation between CNV genes and phenotype variation. Functional enrichment analysis suggests that genes involved in sensory perception of smell, signal transduction, neurological system process and metabolism are affected by the CNVRs. The enrichment of CNVRs involved in the sensory related genes is consistent to the general behavior of pigs, showing strong reliance on their sense of smell in various behavioral contexts. Collectively, this data might assist future studies on some of the genetic variation influencing morphological, behavioral and physiological traits in pigs.

Genes involved in immune response such as interferon (*IFN*), cytochrome P450 (*CYP*), are usually fast evolving due to their importance for the organism to respond rapid changes in the environment. Our results show that these type of genes are often found to be CN variable in pigs. For example, members of interferon (*IFN*) gene families, involved in defense against viral infections, and *CYP* genes, which are responsible for detoxification and drug metabolism, were found to be CN variable. Olfactory receptor (*OR*) represents another gene family that is over-represented in our list of CN variable genes. *Sus scrofa* have the largest repertoire of functional *OR* genes in mammals (from mammals whose genome has been sequenced to date) [33], likely related to the strong dependence on their sense of smell for foraging [25]. Nearly one-third of the 1301 porcine OR genes are found as copy number variable in pigs. These findings suggest that the wide variety of environment faced by pigs around the world resulted in CNVs.

Among defense related copy number variable genes, *NPG3* (from 4 to 23 copies) and *PMAP23* (from 2 to 13 copies) are cathelicidin related porcine specific genes. *NPG3* is responsible for microbicidal activity against *Escherichia coli*, *Listeria monocytogenes* and *Candida albicans in vitro*[45] whereas *PMAP23* exerts antimicrobial activity against both gram-positive and gram-negative bacteria *in vitro*[46]. In addition, *CAMP* (from 3 to 16 copies), another cathelicidin related gene present in the list of copy number variable genes. The observed variation in copy number of cathelicidin related genes suggests an ongoing process of evolution of this gene-family in porcine genome to combat food-borne pathogens.

In humans, copy number of amylase genes, especially *AMY1*, shows high variation between populations (from 2 to 15 copies). High copy number of *AMY1* allows more efficient breakdown of starch [47]. Unlike in humans, pigs have a universally high number of copies (from 8 to 21 copies) of amylases (*AMY1*, *AMY2A*, *AMY2B*) between all individuals, suggesting universal importance of amylases for digesting starch-rich food in this omnivorous species.

Genes such as *BTN1A1* and *F5* are found to be involved in the regulation of milk lipid droplets [48] and preterm delivery in human [49], respectively. Interestingly we found that these genes had variable numbers of copies in different pig breeds. Specifically, Asian breeds have typically a higher number of copies of these genes. In the pig breeding industry, Asian breeds are famous for being highly prolific; with some breeds typically bearing more than 15 live young per litter. These results suggest that these genes have been important in the selection process for highly fertile breeds in Asia. It is notable that some of these fertility genes have high CN in some European breeds (especially Large whites). Recent studies shown that this particular breed has been extensively admixed with Chinese pigs in order to improve fertility traits during the industrial revolution [24,25]. Thus, this pattern could also be the result of this well-known admixture.

Some members of the uridine diphosphate glucuronosyl transferases (UGTs) superfamily are found variable in copy number. UGTs are part of important metabolic pathways responsible for the detoxification and elimination of many different endobiotics and xenobiotics [50]. The *UGT2B10* gene, which is one of the most important genes involved in N-glucuronidation of nicotine, has a higher copy number in Asian individuals (from 5 to 9 copies) than the European individuals (3 copies). The elevated copy number may be related to the ability to detoxifying specific plant secondary metabolites. Although, at present there is no data on wild boar feeding habits in relation to floristic differences between East and West Eurasia, our finding can direct future ecological studies on that subject.

### Demography shape CNVR diversity

Regardless of their geographic origin, different pig populations have undergone different selective pressure. Important events were the foundation of modern pig breeds starting around 200 years ago during the industrial revolution, and more recently, the development of modern breeding practices in the past five decade in different parts of Asia and Europe.

The association of CNVRs with distinct phenotypic effect and different selective regimes in Europe and Asia, suggest that differences in structural variation between wild and domestic pigs as well as Asian and European populations, could reflect domestication history. By including different pigs from the two independent domestications together with individuals representative of their wild ancestors, enabled a first preliminary insight into the change in pattern of CNVRs influenced by the process of domestication and/or the natural demographic history of pigs.

To investigate the importance that CNVRs may have had on phenotypic diversification in breeds, we compared the amount of CNVRs in domesticated and wild individuals. We found more CNVRs in domesticated animals (2,915) than in wild boars (2,879). Moreover, our results showed that CNVR counts were also higher in Asian pigs (combined wild and domestic) (2,967) than in European pigs (2,779) (combined wild and domestic) (Figure 4), which is consistent with a large effective population size and diverse origin of Asian pigs [23,25].

A recent study based on SNPs identified a similar pattern not only between breeds and wild but also between Asian and European pigs [25]. Thus, CN seems to be more variable in larger populations, following the similar patterns as other types of variation such as SNPs [25] and microsatellites [23]. This indicates that the general pattern of CNV is more reflecting demography rather than phenotypic diversity. Having large fractions of common CNVRs between different groups and excess of CNVRs (2,664; 85.43%) in non-genic regions suggest that a major part of these variations are likely to be neutral or nearly neutral. This further supports their reflection on demography rather than phenotypic diversity. These results are of importance as they show that intensive artificial selection did not affect the overall diversity of CNVRs in domestic pigs and do not appear to be the major source of the large phenotypic diversity observed in domestic pigs.

## Conclusion

We identified 3,118 CNVRs with an average size of 13 Kb comprising 39.2 Mb of the porcine genome, which represents the largest source of genetic variation identified in the porcine genome to date. The inferred CNV regions include 545 genes providing an important resource for future analyses on phenotypic variation in pigs. Functional analyses revealed CNVRs enriched for genes related to sensory perception, neurological process, and response to stimulus in specific breeds or wild population. Comparison between wild and domestic groups shows that, beside few exceptions, domestication did not lead to a change in CNVRs among breeds. Moreover, we found that most CNVRs ascertained in domestics were also variable in wild boars. This result suggests that the majority of CNVRs were already segregating among wild boars before domestication. Furthermore, while we identify few CNVRs that may be under selection during domestication and may lead to phenotypic differences, the majority of variable regions were found to reflect demographic pattern rather than selective regimes. Our study represent a comprehensive analysis of CNV in both domestic and wild pigs and provides valuable insight in the evolutionary dynamics of copy number variation, in the context of adaptation and domestication.

## Methods

### Database

In total 16 different individuals originated from 13 populations of *Sus scrofa* were sequenced at different sequencing centers using the Illumina HiSeq platform. The libraries are 100 bases pair-end reads with coverage per animal ranging between 7 – 11×. The sampled pigs comprised of three European wild boars (2- Dutch and 1- Italian), five European domestics (2- Large whites and 1- from each Landrace, Duroc and Pietrain), three Asian wild boars (1- North Chinese, 1- South Chinese and 1- Japanese) and five Asian domestics (2- Meishan, 2- Xiang and 1- Jianquhai) (Table 1; Additional file 1: Table S1A). DNA samples were obtained from blood samples collected by veterinarians according to national legislation or from tissue samples from animals obtained from the slaughterhouse or in the case of wild boar from animals culled within wildlife management programs.

### Sequence alignment and copy number estimation

Copy number of regions in the genomes of all the 16 individuals was detected by the read depth (RD) method [26,34], where the number of copies present is inferred from sequence depth of whole genome sequence data. To calculate the average read depth from those libraries, reads were aligned to the available pig reference genome (*Sus scrofa* build 10.2) using mrsFAST v2.3.0.2 (“Micro-read (substitutions only) fast alignment and search tool” [29]) with an edit distance of at most 7. mrsFAST is a memory efficient and fast software, which reports all possible mapping locations (not only the best, unique or first mapping locations as several other softwares), which is essential in order to detect multi-copy regions using read depth method. Because the RD methods do not take paired end information into consideration, all the paired end libraries were treated as single end libraries.

Highly repeated elements are the main source of noise for the RD method. The porcine genome consists of more than 40 percent of highly repeated elements and most of these repeated elements are long/short interspersed nuclear elements (LINEs/SINEs), long terminal repeats retro-transposons (LTRs) and satellites [25]. To avoid noise from these repeated elements, a repeat masked reference genome was used. Repeat masked information was obtained from NCBI (http://ftp.ncbi.nih.gov/genbank/genomes/Eukaryotes/vertebrates_mammals/Sus_scrofa/Sscrofa10.2/Primary_Assembly/assembled_chromosomes/FASTA/) and merged with the repeat masked information used in Groenen *et al*. (2012) [25]. Calculation of read depth across the whole genome was done with the help of SAMtools v0.1.12a [51]. Average read depth for each 1 Kb non-overlapping bin was calculated across the genome.

RD method uses read depth information of diploid region to infer copy number of each 1 Kb non-overlapping bin present in the genome. No prior information regarding diploid regions in the porcine genome was available. We therefore used 1:1 orthologous genic regions between human, cow and pig as diploid region in the first stage to identify CN of each bin present in the genome (Additional file 3: Figure S1). Since, coding regions are known to have a higher G/C content than an average region of a genome [52,53] this procedure may introduce a G/C biased read depth. To reduce possible G/C bias caused by the 1:1 orthologous regions, all diploid regions predicted from 1:1 orthologous regions in the first stage were subsequently used to calculate the average diploid read depth of the porcine genome (Additional file 3: Figure S1).

Next generation sequencing methods have been shown to introduce a bias in the coverage in regions of high or low G/C. One of the major reason for GC bias coverage in Illumina sequences originates from the polymerase chain reaction (PCR) amplification step during library preparation as well as for cluster amplification on the Illumina flowcell [54]. This issue is similar for any sequencing technology that relies on PCR amplification [55]. To correct for this bias we calculated G/C intervals correction factors as described by Sudmant *et al*. (2010) [26]. These factors were used to correct read depth of each 1 Kb bin across the genome. CN of each 1 Kb non-overlapping bins were then estimated based on the G/C corrected read depth. Since the samples include both male and female individuals, copy number of male X chromosomes were corrected by multiplying the read depth by 2 (outside the pseudo-autosomal regions) to make them comparable with female individuals.

### Prediction of MCRs and defining CNVRs

All the 1 Kb bins with minimum CN of 1 were extracted from all 16 individuals and bins with CN >3 were chained to form multi copy regions (MCRs). The same MCRs might be assigned with different boundaries in different individuals due to technical and/or biological reason and therefore all the MCRs from all individuals were extracted merged and the CN of those regions for all 16 individuals were compared. Copy number variable regions were identified based on the standard deviation of the CN of MCRs in all 16 individuals. Hence, CNVR status was assigned to those regions, which were variable (s.d. ≥0.7) in CN across all 16 individuals.

### Gene identification and gene ontology

All the annotated porcine genes from *Sus scrofa* build 10.2, Ensembl release 67, were extracted using Biomart [56] and genes which were overlapping with the CNVRs (≥70% overlap) were identified. To reduce false calls of particular genes as being multi copy genes, exons of genes overlapping with CNVRs were tested for average CN. GC correction on the read depth of all exons was performed using the correction factors obtained previously for the whole genome. All the genes with an average depth in exon regions >2 were kept in the list of genes affected by CNVRs for further analysis. Not all pig genes have associated gene names, thus the genes without gene names were blasted against the human Refseq mRNAs and human reference protein sequences (blastn and blastp respectively) and the best human hit was assigned as gene name. Human orthologs of porcine genes were used to perform gene ontology analysis. BinGO v2.44 [57] a plugin of Cytoscape v2.8.3 [58] was used to identify enriched GO terms using human gene annotation as background. Hypergeometric test was used to assess the significance of the enriched terms and Benjamini and Hochberg correction was implemented for multiple comparisons.

### Comparison between different groups

For the group comparison, we formed groups based on their geographical location and population type (Asian wild, Asian domestic, European wild and European domestic). To make all the groups comparable with each other, we took 12 instead of all 16 individuals i.e. three pigs per group (Additional file 1: Table S1B). CNVRs for all groups were generated based on the similar approach we used before but instead of 16 individual we compared only individuals present in the particular group.

### qPCR validation

Primer3 webtool http://frodo.wi.mit.edu/primer3/ was used to design primers for qPCR validation. Amplicon length was limited between (50 bp – 100 bp) and regions with GC percentage between 30% and 60% were included, while avoiding runs of identical nucleotides. All other settings were left at their default. Details of the qPCR primers can be found in Additional file 4: Table S4C. qPCR experiments were conducted using MESA Blue qPCR MasterMix Plus for SYBR Assay Low ROX from Eurogentec, this 2× reaction buffer was used in a total reaction volume of 12.5 μl. All reactions were amplified on 7500 Real Time PCR system (Applied Biosystems group). The copy number differences were determined by using a standard ΔCt method that compares the mean Ct value of the target CNV fragments, determined from different input concentrations, compared to the mean Ct value of a known diploid reference.

## Competing interests

The authors declare that they have no competing interests.

## Authors’ contributions

OM, YP, H-JM, MAMG conceived and designed the experiments. YP, OM performed the experiments and analyzed the data. MAMG RPMAC contributed reagents/materials/analysis tools. YP wrote the manuscript. YP, OM, H-JM, LAFF, MB designed and improved pipeline for CNV detection. OM MAMG H-JM LAFF MB JWMB RPMAC discussed and improved manuscript. All authors read and approved the final manuscript.

## Supplementary Material

Additional file 1: Table S1A List of individuals with MCRs. **Table S1B.** List of individuals used to form groups. Click here for file

Additional file 2: Table S2A List of the CNVRs in the porcine genome. **Table S2B.** CNVRs distribution in the porcine genome. **Table S2C.** G/C percentage of the chromosome and region affected by CNVRs. Click here for file

Additional file 3: Figure S1 Flow chart of the MCR detection process. A) First step: Detection of diploid region using 1:1 orthologous region. B) Refining step: Detection of MCRs using the predicted diploid region from the first step. **Figure S2.** The region in chromosome 1 with *SAL1 gene*. The region containing *SAL1 gene* (SSCI: 284447110-284451960) shows high copy number in some of the Asian individuals and European Domestics. **Figure S3.** A CNVR in chromosome 3 (SSC3: 22313001-22324000). A non-genic CNVRs with variable CN between different individuals (higher copy number in European wild boar(CN 7-14) where as some European domestics have CN less than 4). Click here for file

Additional file 4: Table S4A Gene ontology using BinGO package. **Table S4B.** General statistics of genes. **Table S4C.** The list of qPCR primers. Click here for file

Additional file 5: Table S3A Total genes overlapped by CNVRs with their exonic depth. **Table S3B.** Final list of genes overlapped by CNVRs. **Table S3C.** Orthologous genes overlapped by CNVRs. **Table S3D.** List of olfactory receptor genes overlapped by CNVRs. Click here for file
