# Supplementary material for: Evolutionary dynamics of copy number variation in pig genomes in the context of adaptation and domestication
Source: BMC Genomics. 2013 Jul 5;14:449. doi: 10.1186/1471-2164-14-449 (PMC3716681; doi:10.1186/1471-2164-14-449)
Supplement: Additional file 3: Figure S1 — Flow chart of the MCR detection process. A) First step: Detection of diploid region using 1:1 orthologous region. B) Refining step: Detection of MCRs using the predicted diploid region from the first step. Figure S2. The region in chromosome 1 with SAL1 gene. The region containing SAL1 gene (SSCI: 284447110-284451960) shows high copy number in some of the Asian individuals and European Domestics. Figure S3. A CNVR in chromosome 3 (SSC3: 22313001-22324000). A non-genic CNVRs with variable CN between different individuals (higher copy number in European wild boar(CN 7-14) where as some European domestics have CN less than 4). [file 1471-2164-14-449-S3.pdf]

Supplementary Information

Supplementary Figure 1: Flow chart of the MCR detection process.

A) First step: Detection of diploid region using 1:1 orthologous region.

B) Refining step: Detection of MCRs using the predicted diploid region from the first step.

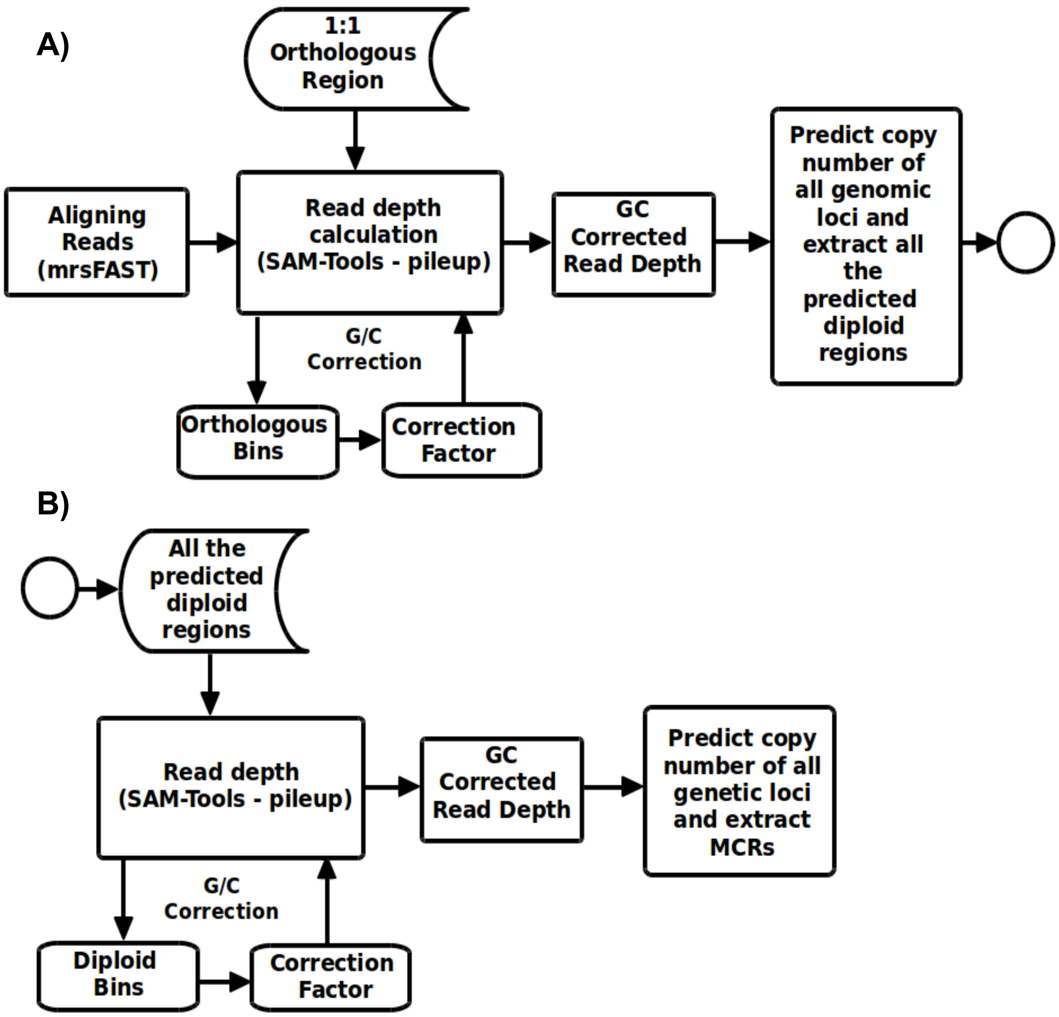

Supplementary Fig 2: The region in chromosome 1 with *SAL1* gene.

The region containing *SAL1* gene (SSC1: 284447110-284451960) shows high copy number in some of the Asian individuals and European Domestics.

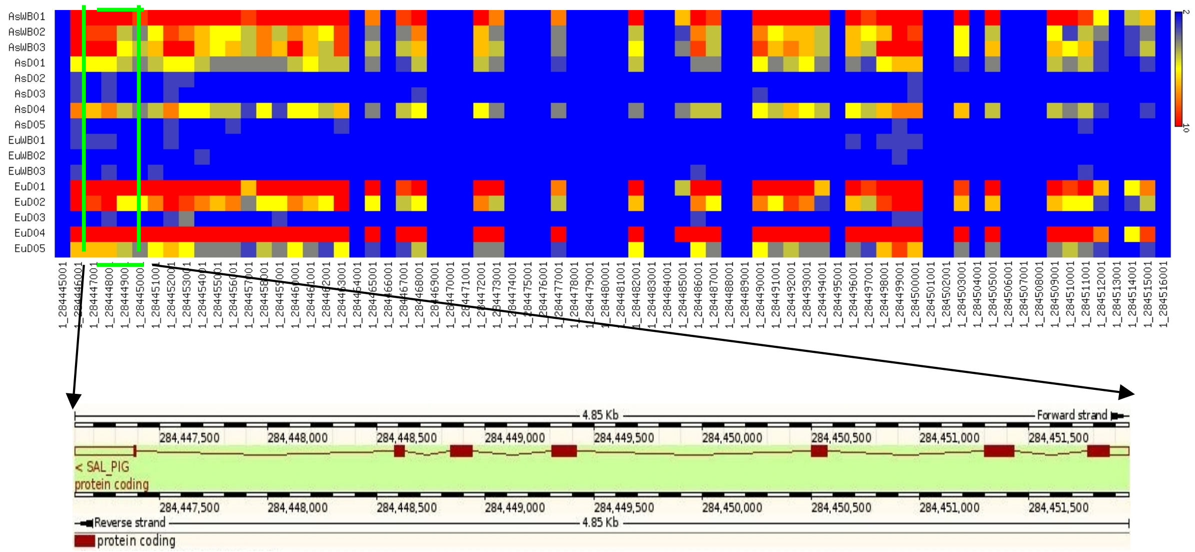

Supplementary Figure 3: A CNVR in chromosome 3 (SSC3: 22313001-22324000).

A non-genic CNVRs with variable CN between different individuals (higher copy number in European wild boar(CN 7-14) where as some European domestics have CN less than 4).

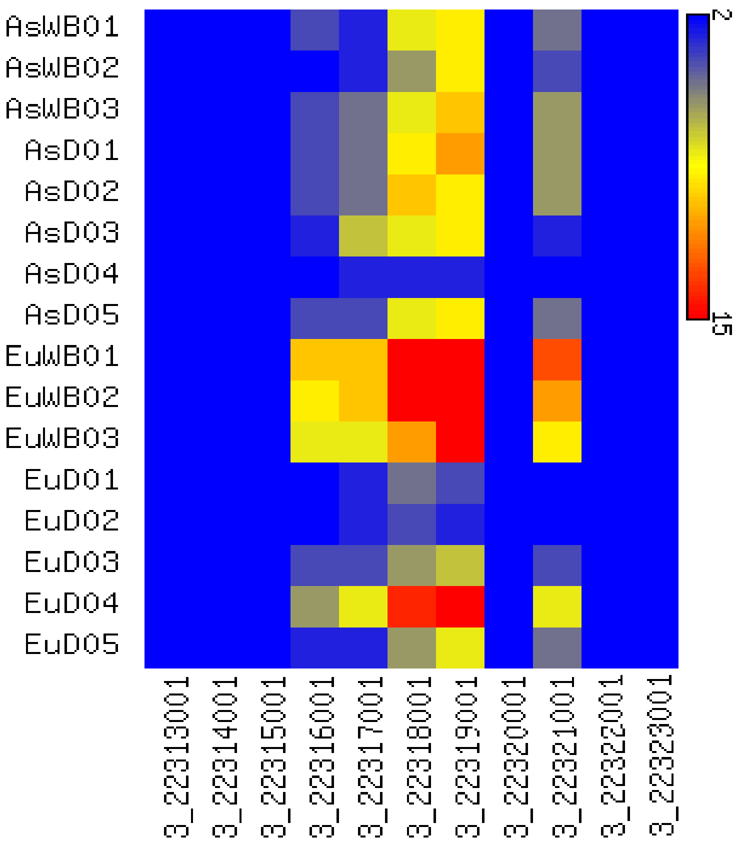

Supplementary Tables

Supplementary Table 1**A**: List of individuals with MCRs

Supplementary Table 1**B**: List of individuals used to form groups

Supplementary Table 2**A**: List of the CNVRs in the porcine genome.

Supplementary Table 2**B**: CNVRs distribution in the porcine genome.

Supplementary Table 2**C**: G/C percentage of the chromosome and region affected by CNVRs.

Supplementary Table 3**A**: Total genes overlapped by CNVRs with their exonic depth.

Supplementary Table 3**B**: Final list of genes overlapped by CNVRs.

Supplementary Table 3**C**: Orthologous genes overlapped by CNVRs.

Supplementary Table 3**D**: List of olfactory receptor genes overlapped by CNVRs.

Supplementary Table 4**A**: Gene ontology using BinGO package.

Supplementary Table 4**B**: General statistics of genes

Supplementary Table 4**C**: The list of qPCR primers

A,B,C,D,E represents sheets in each excel file.
